# Quo Vadis Radiotherapy? Technological Advances and the Rising Problems in Cancer Management

**DOI:** 10.1155/2013/749203

**Published:** 2013-05-16

**Authors:** Barry J. Allen, Eva Bezak, Loredana G. Marcu

**Affiliations:** ^1^Centre for Experimental Radiation Oncology, St George Cancer Care Centre, Kogarah, NSW 2217, Australia; ^2^Department of Medical Physics, Royal Adelaide Hospital, North Terrace, Adelaide, SA 5000, Australia; ^3^School of Chemistry and Physics, University of Adelaide, Adelaide, SA 5000, Australia; ^4^Faculty of Science, University of Oradea, 410087 Oradea, Romania

## Abstract

*Purpose*. Despite the latest technological advances in radiotherapy, cancer control is still challenging for several tumour sites. The survival rates for the most deadly cancers, such as ovarian and pancreatic, have not changed over the last decades. The solution to the problem lies in the change of focus: from local treatment to systemic therapy. The aim of this paper is to present the current status as well as the gaps in radiotherapy and, at the same time, to look into potential solutions to improve cancer control and survival. *Methods*. The currently available advanced radiotherapy treatment techniques have been analysed and their cost-effectiveness discussed. The problem of systemic disease management was specifically targeted. *Results*. Clinical studies show limited benefit in cancer control from hadron therapy. However, targeted therapies together with molecular imaging could improve treatment outcome for several tumour sites while controlling the systemic disease. *Conclusion*. The advances in photon therapy continue to be competitive with the much more expensive hadron therapy. To justify the cost effectiveness of proton/heavy ion therapy, there is a need for phase III randomised clinical trials. Furthermore, the success of systemic disease management lies in the fusion between radiation oncology technology and microbiology.

## 1. Introduction

The AACR Cancer Progress Report (2011) shows that, in the USA from 1990 to 2007, death rates from all cancers dropped 22% in men and 14% in women. More than 68% of adults live five years or more after diagnosis, up from 50% in 1975. For all pediatric cancers, the five-year survival rate is 80%, compared with 52% in 1975. However, the poor survival rates from the most deadly cancers: pancreatic, ovarian, and glioblastoma multiforme (GBM) have not changed to this date.

Keyhole and robotic surgery are major developments in the surgical management of cancer. Patients suffer reduced hospitalisation, but have concomitant costs decreased? Patient quality of life has undoubtedly improved, but are patients living longer?

Pediatric leukaemia is no longer a death sentence and testicular cancer responds well to cisplatin therapy. But most chemotherapy applications are palliative in intent and associated with complications and reduction in the quality of life.

**Figure 1:**
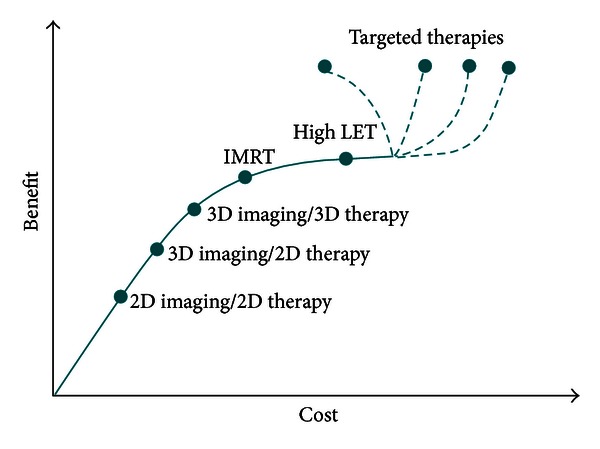
Cost-benefit diagram of current radiotherapy modalities.

External beam radiotherapy has developed over 100 years from low energy X-rays, through Cobalt-60 gamma rays to linear accelerators producing ever-increasing photon and electron energies. Nowadays, external photon beams can be delivered to precise, irregular targets via many modalities: three-dimensional conformal (3DCRT), intensity modulated (IMRT), and image-guided (IGRT) radiotherapies. As excellent as these modalities are, they are directed not so much to the cancers that cannot be controlled but to those that can be. As such, the local control and survival achieved with these technologies do not correlate with the increasing cost of external beam radiotherapy (EBRT) (Figure 1). Still, advances in photon radiotherapy continue to be competitive with the much more expensive high energy proton and heavy ion therapy, used for their exceptional advantage in sparing critical tissues. 

**Figure 2:**
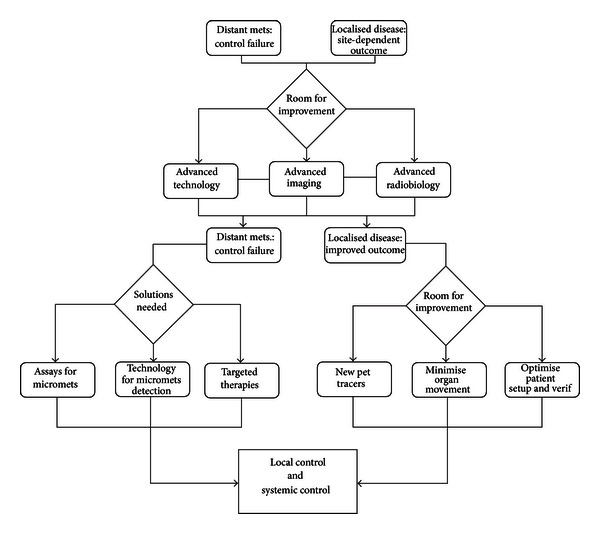
Building blocks of cancer management.

On the imaging side, the development of computed tomography (CT), single photon emission tomography (SPECT), positron emission tomography (PET), magnetic resonance imaging (MRI), and functional MRI (fMRI) has been of immense importance in developing our ability to see into our bodies and examine function. Tumours can be identified down to a few mm in diameter with MRI and to 4-5 mm for functional images with PET. Current in vivo imaging techniques continue to resolve smaller and smaller tumours but can never resolve subclinical disease, by definition, nor tell us where micrometastases lie (see Figure 2).

Without doubt, the oncologist's job of managing cancer has been made much easier and more certain, but has this translated into improved prognoses for cancer patients? The evidence presented by the AACR suggests that the answer is in the affirmative, although early detection may be responsible for much of the improvement. External beam radiotherapy has improved markedly in terms of targeting well-defined volumes and achieving local control but can never eliminate systemic disease. 

The “war on cancer” analogy is often used. But like the 21st century wars, the apparent initial success is masked by the long-term failure to control systemic disease. Few primary cancers are fatal, GBM being the standout, and it is not the primary tumour that determines outcome. It is the cancer cells released from the tumour that invade the vascular and lymphatic systems that eventually lead to multiple metastases and organ failure as normal functional tissues are crowded out by the ever populating cancer cells. Therefore, disseminated disease is the primary cause of cancer death. Yet this objective has been overlooked during the development of modern radiotherapy techniques that are focused on external beam radiotherapy.

On top of this is the rapidly developing cost of the management of cancer. Cancer causes the highest economic loss of all of the 15 leading causes of death worldwide. The World Health Organisation (WHO) note that the economic toll from cancer is nearly 20 percent higher than heart disease, the second leading cause of economic loss ($895 billion and $753 billion, resp.). Health budgets in developed countries are continually expanding and constrained by limited resources. But cost-benefit analyses to determine whether the increased cost of treatment achieves improved outcomes remain unknown. Perhaps the classic example is in the treatment of primary prostate cancer where surgery, brachytherapy, external beam photon, and proton therapies are used, all achieving similar efficacy and complications but at very different capital and running costs.

The developed nations' investment in cancer research has been growing exponentially, and the 2012 budget for the US National Cancer Institute alone will be totalling nearly 6 billion US dollars [1, 2]. 

For rural populations, cancer is often diagnosed at the symptomatic stage. Palliative therapy is the only course to reduce pain and increase quality of life. As a result, there is a need to provide low cost technologies appropriate for the objective of palliation. Because of the continuing shortage of trained oncologists, medical physicists, and other professionals, telemedicine must be an essential element in the development of radiotherapy care in rural regions. 

We are therefore confronted with two distinct challenges:

1. to manage and control systemic cancer,
2. to provide cost-effective technologies.

## 2. Current Status and Gaps

### 2.1. External Beam Radiotherapy and Intensity Modulated Radiation Therapy

EBRT and IMRT are the current standards of care for radiation oncology patients. IMRT is known to be a highly conformal treatment, involving reduced treatment margins which results in improved tumour control and reduced normal tissue toxicity. However, the ability to deposit a high dose into well-defined tumours is not always desired, as too precise targeting can lead to increased local recurrences. The biology of the tumour must determine the clinical tumour volume (CTV) margin, not the resolution of beam delivery. If the CTV margin cannot be fully imaged, then this becomes the most difficult margin to be determined. The decision is typically based on clinical assessment of risk, on local clinical practice, and on historical evidence rather than the tumour spread quantified in an individual patient. Often a uniform CTV margin is used regardless of potential anisotropy in the microscopic tumour spread. The extent of the CTV margin depends on information received from imaging techniques. Such accurate, individualised CTV determinations represent one of the problems where developments in diagnostic imaging (molecular imaging techniques in particular) will improve radiotherapy treatment [3].

Other factors include problems with treatment times and dose gradients. IMRT increases the volume of normal tissue exposed to a low dose. However, what might have been thought to be a negative factor could be positive, in view of hormesis effects.

### 2.2. Hadron Therapy

Protons offer greatly improved and precise dose deposition and as a result lower normal tissue doses. But does proton therapy always give superior efficacy, reduced complications, improved prognosis, and lower cost therapy? While there are unique applications of proton beams that give reduced adverse events, especially for pediatric cancers [4, 5], the answer appears to be negative. Prostate cancer is a major application for proton machines. But prognosis for prostate cancer is mostly set not by the primary therapy but by the initial stage of the disease on diagnosis [6, 7]. As such, low risk patients with PSA < 10 and Gleason 6 are very unlikely to succumb to the disease and will not be in need of radical therapies. 

Uveal melanoma (an extremely low incidence cancer) was a long-standing application for proton therapy. However, on the basis of an intralesional skin melanoma trial of targeted alpha therapy [8], there is every reason to think that intralesional injection into the uveal melanoma will not only save the eye but could well eliminate the micrometastases arising from the primary site that lead to patient death within 5–10 years.

There is increasing evidence that improved control of the primary lesion, arising from the new modalities for protons and heavy ion (C-12) beams, translates into improved survival [9, 10]. However, care must be taken to distinguish between survival and progression-free endpoints, which are not necessarily correlated. 

### 2.3. Cost Benefit of Proton, Heavy Ion, and Photon Therapy

The major advantage of IMRT is its low capital and running costs. While patient charges are not so much of an issue for a well-insured patient, there is a major health issue relating to the availability of radiotherapy to cancer patients. Does western society need many more IMRT units or one proton therapy facility only? Also, is proton therapy cost-effective and is there evidence in clinical trials supporting clinical gains of proton therapy versus conventional therapy? If the current evidence-based medicine standards are used for determination of clinical efficacy of proton therapy, it must be concluded that the evidence is limited and mostly based on noncontrolled studies. A Phase III clinical randomised trial has not been conducted for proton therapy.

Several studies have been though conducted trying to identify the cost-benefit of proton therapy and the type of cancer patients that might benefit from proton therapy [11, 12]. Several investigations concluded that for approximately 15% of cancer patients treated with radiation therapy, proton therapy might be cost-effective. According to Glimelius and Montelius, “This means that one proton therapy facility being able to yearly treat a reasonable number of patients (1000–2000) is motivated for a western world population of about 10 millions. The same estimations for light ions, however, much more uncertain, came to an estimate of 50 millions” [13].

Lodge et al. [14] conducted a systematic literature review (comprising, 54 publications: 4 randomized controlled trials reported in 5 publications, 5 comparative studies, and 44 case series) on the efficacy of hadron therapy (therapies including protons, neutrons, and light and heavy ions), concentrating on external beam techniques. They found that there is insufficient clinical evidence available to justify the rapid expansion of hadron facilities as a major treatment modality and recommended that further research into the clinical and cost-effectiveness of hadron therapies was required. The authors concluded, “the current literature shows that the introduction, or significant extension, of hadron therapy as a major treatment modality—except on a minor scale for certain rare tumours (ocular, chordomas, etc.)—into standard clinical patient care cannot be supported by the evidence base currently available. There are little reliable evidence-based data available concerning the relative cost-effectiveness of hadron therapy interventions when compared with each other, with photon therapy, or with other cancer treatments.” 

### 2.4. Targeted Therapy

Although not a standard of care in radiation oncology, targeted therapies are emerging treatment techniques with the ability to control systemic disease. With advances in immunology and the development of exquisite targeting vectors, we can now engage in targeted imaging and therapy using the alpha, beta, and gamma rays emitted from radioisotope labels. However, the use of radioisotope therapy, apart from the thyroid and palliative therapy for bone cancer, has had limited effect on survival. Beta emitters have limited efficacy in the control of cancer, although new radioisotopes are finding niche applications with various targeting agents. Much more successful has been the use of gamma ray emitters for imaging with SPECT and PET.

The next generation of radioimmunotherapy may lie with alpha-emitting radioisotopes that can selectively kill targeted cancer cells. The field of targeted alpha therapy is slowly gathering momentum, with some 10 clinical trials completed, in operation, or at the planning stage around the world. This fusion of biology and medical physics suggests that we should use the term biomedical physics much more than we do.

### 2.5. Tumour Site-Specific Treatment Challenges

The future objectives for radiation therapy have to address the failures of current therapies. Some of the site-specific treatment challenges encountered by today's cancer management are mentioned below.

- *Primary prostate cancer*: there is the need to spare the nerves and rectum to reduce incontinence, impotence and rectal perforation.
- *Lung cancer*: tumours can be regressed but there is the need to eliminate subclinical metastases.
- *Glioblastoma multiforme*: it is fatal in the primary state, we need to improve prognosis by eliminating subclinical disease in the brain by extravascular delivery.
- *Metastases to the brain*: they are invariably a fatal stage of cancer which requires elimination of subclinical metastases by vascular delivery.
- *Uveal melanoma*: primary treatment needs to be integrated with inhibition of lethal liver metastases.
- *Advanced head and neck cancer*: it has poor outcome despite the multidisciplinary treatment approach. Around 80% of head and neck cancer patients overexpress the epidermal growth factor receptor (EGFR) which is linked to poor prognosis. Targeted molecular therapy against EGFR could play a pivotal role as adjunct therapy.
- *Pancreatic cancer*: it is fatal in the primary state due to late detection. There is a need to improve prognosis by targeted immunotherapy against EGFR. While patients with mutated EGFR might benefit from Erlotinib (an EGFR inhibitor), the side effects limit the efficacy of the drug. Tumour infiltration and liver failure are ultimately the fatal stage. Another approach is to use systemic alpha therapy against uPA and/or MUC1 receptors [15].
- Pretesting for patient radiosensitivity could avoid overdosing of *normal tissue* in sensitive patients and underdosing of the tumour in insensitive patients.

These problems relate to systemic cancer therapy as well as local therapy. Improved spatial dose resolution is important for tumours, but it is the ability to kill subclinical cancer cells that has become the issue. The main problems to be solved regarding systemic cancer therapy are summarised as follows.

- Micrometastases may be in the G0 phase and outside the cell cycle; as such these cells are insensitive to chemo- and radiotherapy and are best treated by high LET radiation.
- Normal tissue radiosensitivity for high LET radiation needs to be investigated.
- Systemic agents must target cancer cells via the vascular and/or lymphatic systems. The tumour capillary permeability becomes an important determinant as to whether the targeting agent can diffuse into the extravascular space.
- Monitoring of systemic therapy via sequential peripheral blood analyses of circulating cancer cells.
- Tumour dormancy and factors responsible for inter-patient variability. The mechanisms behind cancer dormancy need to be elucidated and therapeutic targets identified. The state of dormancy needs to be indicated by biomarkers to predict outcome.

## 3. Current Solutions and Their Limitations

Improved treatment efficacy and/or reduced normal tissue toxicity could arise from simple changes to protocols or to complex systemic therapies. Advances in hadron therapy, targeted and molecular therapies, and well-designed predictive assays for both tumour and normal tissue might be the answer to the problems raised above.

### 3.1. Hadron Therapy

#### 3.1.1. Protons and Heavy Ions

The biophysical and radiobiological properties of heavy ions render them suitable for the management of complex anatomical structures and radioresistant tumours. For example, unresectable osteosarcomas are good candidates for proton/carbon ion treatment as the dose necessary for curative radiotherapy is too large to be deliverable with photon therapy. Also, these tumours are often located in close proximity to radiosensitive organs such as the brain; spinal cord, or pelvis, therefore conventional treatment is not suitable for their management [16].

The Japanese research group from the Research Centre for Charged Particle Therapy National Institute of Radiological Sciences, Chiba [17–19], have demonstrated the efficiency of carbon ion radiotherapy on several tumour sites and types such as locally advanced head and neck tumours; early stage NSCLC and locally advanced NSCLC; unresectable locally advanced bone and soft tissue sarcomas; locally advanced hepatocellular carcinomas; locally advanced prostate carcinomas; chordoma and chondrosarcoma of the skull base and cervical spine; malignant gliomas and oesophageal cancer.

While protons do not offer clinical advantage to certain tumour sites when compared to photon treatment, for ocular tumours Lodge et al. concluded that proton therapy results in approximately 90% eye preservation rates and 50% vision preservation after 5 years. The clinical superiority of protons in ocular radiotherapy was particularly demonstrated for larger tumours and specific locations in the eye, with the benefit dropping off for smaller lesions (e.g., <4 mm). 

Table 1 (modified from [14]) provides a summary of comparative clinical results for proton and ion therapy with conventional photon therapy. In base of skull chordomas, both protons and ions appear to provide superior results to photon therapy. In other central nervous system (CNS) tumours the results of photons were found to be similar to those reported for protons and ions. 

There were no definitive conclusions on the relative cost-effectiveness/gains of protons and heavy ions compared to photons for head and neck cancer, gastrointestinal tumours, non-small cell lung cancer, sarcomas, cancer of the uterine cervix, and bladder cancer. Similarly, for the case of the locally advanced prostate cancer, both photons and protons resulted in similar therapeutic gains. Furthermore, while effective on well-localized tumours, hadron therapy is not a solution for the management of systemic disease.

As of 2010, there are 31 active proton therapy centers, 2 heavy ion facilities, and approximately 12 neutron therapy centers in operation [20]. There are as many as 22 new, planned, or proposed proton therapy centers, some of which are already under construction. In some of these new centers it is proposed to combine both proton and heavy ion therapy facilities. 

The capital cost of a proton therapy facility is around $140 million for a large facility with four treatment rooms, reducing to approximately $50 million for a minimal facility. The cost per patient treatment is around $20–25,000. Standard radiotherapy is exceptionally cheap and perhaps should not be used for comparative purposes. One benchmark is $50,000 per life-year saved, but it is difficult to show that proton therapy actually saves lives. Costs for other therapeutic modalities are surgery ~$15,000, chemotherapy ~$30,000, and bone marrow transplant ~$70,000. However, these modalities are not associated with high capital costs.

#### 3.1.2. Neutrons

Neutrons have been trialled in the past for various malignancies due to their high LET properties. Radioresistant, hypoxic, and/or slowly-growing tumours were found to be good candidates for neutron therapy. Although neutrons can be twice as toxic to hypoxic cancer cells as photons for the same absorbed dose (the oxygen enhancement ratio of neutrons is around 1.6 compared to OER = 3 for photons), the supporting experimental evidence for superior efficiency of neutrons as compared to photons on hypoxic tumours remains inconclusive. Salivary gland tumours and radioresistant sarcomas are probably the only cancers which were shown to benefit from fast neutron treatment. 

Boron neutron capture therapy involving epithermal (for deep seated tumours) or thermal neutrons (for superficial tumours) showed promising results for a limited number of tumour sites such as the brain (glioblastoma multiforme), melanoma, and head and neck [21–23]. While the high RBE of epi/thermal neutrons implies a good outcome, the success of the method depends on the selective uptake of boron compounds by cancer cells. 

In postsurgical glioblastomas the problem arises in the inability to kill cancer cells as they infiltrate in the hyaluronic acid flow through the perivascular space in the brain. For external beam radiotherapy, the therapeutic gain is less than 1, as radiation damages normal tissue cells more than the cancer cells. If high LET radiation is used, any gains from reducing cell cycle effects are lost by delayed radiation necrosis when quiescent endothelial and other cells undergo mitotic cell death. A solution to this problem would be to selectively target the GBM cells and kill them with short range cytotoxicity. BNCT had some success with this process, with a boron-10 compound being taken up by the cancer cells and activated in situ by an external beam of neutrons. However, bioavailability could be a limiting factor in controlling the cancer.

### 3.2. Targeted Therapies

The next major breakthrough in cancer management lies in the fundamental biology and genetics knowledge to address the aforementioned problems rather than further improvements of physical technology which can never reach the required resolution. 

Targeted alpha therapy is a novel high LET therapeutic approach which incorporates the essential elements for cancer therapy: a targeting molecule that fixes to membrane-bound molecules on the surface of cancer cells, and a cytotoxic radiation that deposits a large fraction of energy into the targeted cell. 

While alpha therapy is still a work in progress, developments are being made in translating from preclinical studies to clinical trials. Alpha therapy is demonstrating efficacy in leukaemias as well as in glioblastomas, where results from intracavity administration are very promising, with a 52-week median survival. The use of peptides for targeting GMB is also under investigation [24]. However, the promise of targeted alpha therapy is greatly extended by the development of tumour antivascular alpha therapy (TAVAT) for solid tumours [25]. Metastatic melanoma results show surprising tumour regressions at doses very much below the maximum tolerated dose and, if further research is successful, could change the prognosis for end-stage cancers [26].

### 3.3. Molecular Imaging

Understanding of cancer genetics and biochemistry will improve with advances in molecular imaging, combining physics, chemistry, biology, and technology. In particular, developments in fluorescent imaging enable us to observe many cellular and subcellular processes in vivo. In this technique a fluorescent protein is fused with a cell protein/gene to be investigated. The composite protein/gene is inserted into the cell of interest. The cell gene/protein then performs its function while the attached fluorescent protein identifies the position of the gene within the cell by emitting fluorescent light [27].

The research into the so-called cell penetrating imaging probes (i.e., cell penetrating peptides or CPP) made visualization of biochemical processes in cells possible. These peptides can translocate (through the cell membrane) covalently attached “cargo” (i.e., molecule of interest) into a mammalian cell without requiring specific receptors. 

As for the patient tumour imaging, due to significant absorption of fluorescent light this modality can only be used for very superficial dermatological tumours or tumours/tissues accessible via endoscopy. Another application involves tumour cell illumination during surgery thus enabling more accurate determination of tumour margins. 

The principle of molecular imaging is also applicable to molecular therapy. A large number of different cargo molecules have been successfully delivered inside cells using cell penetrating peptides including proteins, liposomes, and nanoparticles [28]. Peptides have also been developed whose uptake into cells is triggered by enzymes typical of tumours [29]. As a result, the CPPs have potential for targeted/selective delivery of radioactive, magnetic, nanoparticle agents and therapeutic drugs into the cancerous tissues. 

### 3.4. Individualized Treatment Planning

Personalized medicine is the “leitmotiv” of the last decade's oncology. Three major advances are emphasized to overtake traditional patient care in oncology: (1) the development and availability of drugs which inhibit oncogenetic targets; (2) the implementation of advanced technologies to allow for prediction of treatment sensitivity and risk of recurrence; (3) reclassification of malignant diseases with the aim of expanding the number of orphan molecular diseases [30]. Yet, treatment differentiation among patients based on tumour kinetodynamics, metabolism, and radiobiological characteristics remains minimal. 

It is an acknowledged fact that patients with hypoxic tumours can gain from adjunct treatment with hypoxic cell sensitizers or dose “painting” to allow for higher dose delivery with conformal radiotherapy. However, patients with tumours which are better oxygenated should not be exposed to hypoxic cell cytotoxins which inevitably add to the risk of adverse events without any benefit to tumour control. Similarly, those patients that present with highly proliferating tumours were proven to gain from accelerated radiotherapy and cell cycle-specific chemotherapy, whereas patients having tumours with slow cell turnover show clinical advantage when treated with conventionally fractionated radiotherapy and cycle nonspecific chemotherapy. It is therefore important to focus on individualized treatment and to develop predictive assays which can reflect the outcome. 

Biased and inconclusive clinical trial results due to inadequate patient selection have proven the need to consider radiobiological parameters such as hypoxia, proliferative ability, radioresistance, and of other epigenetic factors in their design for more eloquent conclusions. However, predictive assays trialled over the last two-three decades for oxygenation status, proliferative ability, and intrinsic radioresistance showed several limitations and they were never implemented into clinical settings (Table 2). Assays to predict normal tissue response to radiation will always be easier to understand, develop, and integrate in patient care than those targeting the tumour. The challenge to “predict” treatment response is multifaceted making it difficult to establish one complex predictive parameter. There is though potential in tumour parameter-specific radioisotopes used in PET to serve as predictive assays, especially when used in combination to provide supplementary metabolic information and diagnostic specificity (such as FDG + FMISO).

Despite the fact that clinical reports strongly suggest the routine incorporation of functional imaging such as PET/CT in the management of several malignant sites [31–33], there are impediments, whether related to the health system or to physicians' conventionality, which hinder this chance for a better outcome. Besides the accurate staging, tumour-specificity, and predictive ability of PET/CT, employing functional/metabolic imaging techniques, also assists in the early detection and localization of distant metastases. 

However, there are limits to detection set by the tumour size and its metabolism, such that tumours of a few mm diameter, which contain millions of cancer cells, remain subclinical. Further, improved staging improves management, but may not impact on final outcome if the therapy is inadequate. Ultimately, recurrence arises from subclinical disease.

New tumour-specific assays might be needed to complement the existing ones for a better outcome prediction. Immunocytochemical and molecular assays for detection of micrometastases via sequential peripheral blood analyses of circulating tumour cells could add to the monitoring of systemic therapy and to patients' prognosis. Studies into tumour dormancy could answer the question as to why some patients recur soon after primary treatment and others have dormant tumours for long after the completion of therapy? The most commonly accepted explanation for tumour dormancy is the failure of angiogenic switch activation, a state that maintains the balance between proliferation and apoptosis [34]. It was suggested that induction of growth arrest within small groups of tumour cells (cellular dormancy) and immunosurveillance (which prevents residual tumour cell expansion) are other mechanisms behind cancer dormancy [35]. Molecular assays for tumour recurrence and distant metastases are awaited to be developed to scrutinize the anti/proangiogenic balance within the malignant tumour, growth arrest, and adaptive response to a suboptimal microenvironment [36]. Monitoring disseminated and circulating tumour cells is the first step towards better diagnosis. However, the large number of patients that survive with disseminated tumour cells in the bone marrow indicates the need to identify additional molecular factors which characterize the microbiology of disseminated cells [37].

Consequently, the future of targeted cancer treatment to manage disseminated disease probably lies in the ability to induce cell kill of dormant tumour population, to reprogram malignant cells into growth arrest, and to develop biomarkers to indicate the state of dormancy (rather than informing about tumour recurrence).

#### 3.4.1. Normal Tissue Radiosensitivity (RS)

Variable radiosensitivity of individual patient may explain recurrent cancer as well as normal tissue damage in external beam radiotherapy. Radiosensitivity could be a double-edged sword: if patients range from high to low radiosensitivity, then extreme tissue response is seen for the former and poor tumour control in the latter. If the reasonable assumption is made that all cells in the body, including cancer cells, have the same genetic RS, then the cancer would be controlled by lower and safe doses. However, the opposite effect will also be true. Low RS means that higher but safe doses are needed to control the cancer. Pretesting of patient RS may need to be introduced as an important part of the dose planning strategy.

### 3.5. Healthy Tissue Protection

Healthy tissue protection and reduction of treatment-related side effects are other important considerations for the future. Chemotherapy as well as targeted agents needs to be customized, since the same drug and dosage can have minimal normal tissue toxicity in one patient and be lethal to the other. It is suggested that single nucleotide polymorphism (SNP) is a viable technique to be used for the prediction of adverse events on an individual basis and will undergo further research to be clinically validated [30].

Patients have variable radiosensitivity, such that those with high values will be overdosed for normal tissue and those with low values will be underdosed for tumour control. Adjustment to the intrinsic patient's radiosensitivity could be a far more important factor than improved dose delivery systems. As such, radiosensitivity assays need to be developed which could become an essential part of the patient workup before radiotherapy. 

More in-depth studies are needed regarding low doses of radiation and their potential hormetic effect on healthy tissue. Bystander effects induced by low doses of radiation to healthy tissue surrounding the target and the consequential adaptive response to radiation have gained interest and initiated translational research from basic to preclinical studies. There is growing evidence suggesting the potential role of low dose irradiation in clinical settings. Bystander effects are expected to have an impact on systemically targeted therapies; however the limitation of existing dosimetric methods to describe dose-response relationships of various cell lines at low doses makes it difficult to assess the extent of cellular effects outside the treated area.

## 4. Conclusions

The future of radiation oncology technology lies in its fusion with biology. Otherwise, it is hard to see any major developments in the near future with respect to new technologies or even the need for more expensive equipment. The last decades have seen major advances in cancer imaging and external beam therapy, and such investments have already paid off in terms of achieving improved local control. However, they are not matching the current needs in the management of cancer. These needs centre on the control of systemic disease by systemic treatment. Chemotherapy has fallen short here. The fusion of biological targeting and high LET radiation may well be our best hope (although there is little evidence that funding agencies and “peer” reviewers share that opinion). Therefore, biology-driven clinical trials are expected to prevail hand in hand with the development of new targeted agents.

Based on the above-reasoned problems and possible solutions, the focus of future cancer control and management should be on the following.

- TAT: immunotherapy of cancer cells using high LET, short range alphas and TAVAT.
- Knowledge of molecular changes in cancer which can lead to the design of therapies that target proliferation and survival of cancer cells within a tumour as well as in the surrounding tumour microenvironment (i.e., development of genetically informed cancer medicine).
- The complexity of molecular interactions within and among cancer cells, and between cancer cells and normal cells, which will require refinement of multi-agent treatment approaches against cancer.
- Molecular diagnostics: development of tests that will determine mutated genes in a patient's tumour, tumour expressed molecules that can be targeted, and the overall mutational gene expression profiles that can predict a response to the treatment.
- Individualised patient treatments: treatments will have to be modified depending on the molecular profile of a particular tumour. For each patient the optimal match between the tumour and therapeutic regimen will be identified based on biological/genetical information.
- Healthy tissue protection with low doses of radiation by utilizing potential hormesis and bystander effects.

Cancer may be ultimately controlled by genetic antisense techniques that find and cancel out genetic aberrations without causing any complications. However, this ideal objective continues to elude us and, in the meantime, we need to apply less ideal techniques to improve prognosis.

Nevertheless, the majority of the world's populations are a long way from curative cancer. Their needs call for a new paradigm that brings palliative cancer therapy to the rural populations. The cost-benefit of such centers is readily apparent. They can also become centers for cancer screening and ultimately upgraded for curative therapy. 

## Figures and Tables

**Table 1:** Comparative clinical results for proton and heavy ion therapy with conventional therapy (modified from [14]).

Tumour site	Proton therapy		Heavy ion therapy	
	Studies/Patients	Results	Studies/Patients	Results
Head and neck	2/62	Not conclusive	2/65	Not conclusive
Prostate	3/1751	Similar	4/201	Not conclusive
Eye	10/7708	Superior	2/1343	Similar to protons
Lung	3/156	Not conclusive	3/205	Similar to stereotactic radiotherapy
CNS	10/839	Similar	3/405	Similar to protons
Gastro-intestinal	5/369	Not conclusive	2/73	Not conclusive
Pelvic	3/80	Not conclusive	2/49	Not conclusive

**Table 2:** Predictive assays for tumours: past, present, and future.

Primary tumour			Systemic disease
Oxygen status	Proliferative potential	Intrinsic cellular radiosensitivity	Micrometastases tumour dormancy
Past			

(i) Electrodes (ii) Biopsy (vascular density) (iii) Endogenous markers (iv) Exogenous markers	(i) Measurement of Tpot, TS, and LI and correlation with outcome (ii) Adjustment of treatment schedule as a function of Tk	(i) Comet assay (ii) Cell survival curves (iii) Functional assays	??

Present			

(i) Oxygen-specific PET markers (FMISO, FAZA) (ii) BOLD MRI	(i) Proliferation-specific PET markers (FLT)	(i) Functional/genomic assays	(i) Immunocytochemical and molecular assays to detect occult metastatic tumour cells

Future			

(i) Oxygen-specific PET markers with higher specificity (FMISO, FAZA, FETNIM, and F-EF3,5) (ii) BOLD MRI	(i) Proliferation-specific PET markers with higher specificity (FLT, F-ISO-1)	(i) Functional/genomic assays	(i) Immunocytochemical and molecular assays to detect disseminated and circulating tumour cells (ii) Biomarkers to indicate the state of dormancy
